# *Pseudomonas aeruginosa* Quorum-Sensing and Type VI Secretion System Can Direct Interspecific Coexistence During Evolution

**DOI:** 10.3389/fmicb.2018.02287

**Published:** 2018-10-11

**Authors:** Kelei Zhao, Lianming Du, Jiafu Lin, Yang Yuan, Xiwei Wang, Bisong Yue, Xinrong Wang, Yidong Guo, Yiwen Chu, Yingshun Zhou

**Affiliations:** ^1^Antibiotics Research and Re-evaluation Key Laboratory of Sichuan Province, Sichuan Industrial Institute of Antibiotics, Chengdu University, Chengdu, China; ^2^Institute for Advanced Study, Chengdu University, Chengdu, China; ^3^Key Laboratory of Bio-Resources and Eco-Environment (Ministry of Education), College of Life Sciences, Sichuan University, Chengdu, China; ^4^Department of Pathogenic Biology, College of Preclinical Medicine, Southwest Medical University, Luzhou, China

**Keywords:** *Pseudomonas aeruginosa*, quorum-sensing, T6SS, evolution, coexistence, microcommunity

## Abstract

It is reported that a wide range of bacterial infections are polymicrobial, and the members in a local microcommunity can influence the growth of neighbors through physical and chemical interactions. *Pseudomonas aeruginosa* is an important opportunistic pathogen that normally causes a variety of acute and chronic infections, and clinical evidences suggest that *P. aeruginosa* can be frequently coisolated with other pathogens from the patients with chronic infections. However, the interspecific interaction and the coexisting mechanism of *P. aeruginosa* with coinfecting bacterial species during evolution still remain largely unclear. In this study, the relationships of *P. aeruginosa* with other Gram-positive (*Staphylococcus aureus*) and Gram-negative (*Klebsiella pneumoniae*) are investigated by using a series of on-plate proximity assay, *in vitro* coevolution assay, and RNA-sequencing. We find that although the development of a quorum-sensing system contributes *P. aeruginosa* a significant growth advantage to compete with *S. aureus* and *K. pneumoniae*, the quorum-sensing regulation of *P. aeruginosa* will be decreased during evolution and thus provides a basis for the formation of interspecific coexistence. The results of comparative transcriptomic analyses suggest that the persistent survival of *S. aureus* in the microcommunity has no significant effect on the intracellular transcriptional pattern of *P. aeruginosa*, while a more detailed competition happens between *P. aeruginosa* and *K. pneumoniae*. Specifically, the population of *P. aeruginosa* with decreased quorum-sensing regulation can still restrict the proportion increase of *K. pneumoniae* by enhancing the type VI secretion system-elicited cell aggressivity during further coevolution. These findings provide a general explanation for the formation of a dynamic stable microcommunity consisting of more than two bacterial species, and may contribute to the development of population biology and clinical therapy.

## Introduction

It has generally been accepted that bacteria will communicate with the neighboring intra- and/or interspecific individuals in a microcommunity and synergistically contribute to the development of diseases. Therefore, clinical therapy solely targeting the primary pathogen may not always be successful (Little et al., 2008; Peters et al., 2012; Short et al., 2014; Pragman et al., 2016). Bacterial respiratory tract infection is a common clinical disease, and increasing evidences have suggested that a majority of chronic lung infections are polymicrobial (Short et al., 2014; Wunderink and Waterer, 2014; Pragman et al., 2016). A recent clinical investigation on the prevalence of respiratory pathogens from 19 key hospitals across China during the past 10 years shows that, among the 229,170 bacterial isolates, *Pseudomonas aeruginosa* (PA, 16.5%) and *Klebsiella pneumoniae* (KP, 14.8%) are the leading species of Gram-negative (G^-^) bacteria, and *Staphylococcus aureus* (SA, 11.8%) is the dominant Gram-positive (G^+^) bacteria (Yang et al., 2016). The carbapenem-resistant PA and KP are recognized as Priority 1 (critical) antibiotic-resistant bacteria in the latest global priority list released by the World Health Organization (2017), and the methicillin-resistant SA is listed in Priority 2 (high). These species can be coisolated from the samples of pneumonia and burn patients (Liu et al., 2004; Li et al., 2007; Wu et al., 2016; Acosta et al., 2017).

Bacteria normally secrete numerous extracellular products to the local ecosystem for nutrient acquisition and population fitness, and the elaborate quorum-sensing (QS) system that is activated upon the recurrently binding of signal molecules at a high cell density can significantly contribute to these processes (Fuqua et al., 1994; Waters and Bassler, 2005; Asfahl and Schuster, 2017). The QS system of PA has been well characterized. LasR is the central regulator of the QS hierarchy and the *las-rhl* regulatory cascades can trigger the expression of most exoenzyme- and virulence factor-encoding genes (Schuster et al., 2003). These costly extracellular products can be freely used by other individuals in the pool and thus establish a basis for bacterial social cooperation (Diggle et al., 2007; Xavier et al., 2011; Asfahl and Schuster, 2017). However, this cooperative interaction is susceptible to the invasion of QS-deficient individuals (especially the *lasR* mutants) and may lead to population divergence, which frequently happens during *in vitro* evolution and in causing chronic lung infections (Smith et al., 2006; Sandoz et al., 2007; Köhler et al., 2009; Wilder et al., 2011).

Competition is considered as the mainstream of cell–cell interactions among microbial species (Foster and Bell, 2012). The wild type (WT) can efficiently suppress the growth of SA by exerting robust QS ability (Korgaonkar et al., 2013; Hotterbeekx et al., 2017). By contrast, the host-adapted PA isolates with a deficient QS system show a commensal-like interaction with SA (DeLeon et al., 2014; Frydenlund Michelsen et al., 2016). Additionally, PA can kill heterogenous G^-^ competitors by employing the type VI secretion system (T6SS) to inject a variety of protein toxins (Hood et al., 2010; Russell et al., 2014). Three distinct T6SSs (H1, H2, and H3), which are classified according to the locus of the hemolysin-coregulated protein secretion island, have been identified in the genome of PA (Mougous et al., 2006), and the performances of these T6SSs are also implicated in the regulation of the QS system (Schuster et al., 2003; Lesic et al., 2009). Therefore, based on the fact that different kinds of bacterial pathogens can be coisolated with PA from clinical samples, we are interested to find out how PA can solve the problem of interspecific competition and coexist with other species, and to probe the potential roles of the QS system and T6SS of PA during the development of the microcommunity. In this study, to provide a general explanation for the coexistence of PA with other G^+^ and G^-^ bacteria, the model PA strain (PAO1) was used to explore the relationships of PA with SA and KP by performing a series of coculture assays and RNA-sequencing (RNA-Seq). Although PA was found to be the dominant species, SA and KP could coexist with PA during evolution and the competition among the three pathogens mainly happened between PA and KP. The result of comparative transcriptomic analyses further revealed that the decreased QS regulation and enhanced T6SS of PA could contribute to the formation of PA-dominated microcommunity.

## Materials and Methods

### Bacterial Strains, Plasmids, and Media

Strains of PA (*P. aeruginosa* PAO1), SA (*S. aureus* ATCC 25923), KP (*K. pneumoniae* ATCC 700603), and their derivatives and plasmids used in this study are listed in **Supplementary Table 1**. Clinical isolates of PA (SWM2 and SWM21), *Corynebacterium argentoratense* (CA) and *Escherichia coli* (EC) were coisolated from the deep part sputa of a patient (85–90 years old) who suffered from chronic obstructive pulmonary disease (COPD) for over 20 years. Species identification was performed by DNA sequencing of 16S rDNA. SWM2 showed a PAO1-similar phenotype, while SWM21 harbored a *lasR* loss-of-function mutation at nucleotide site + 455. All the strains were routinely cultured in Luria-Bertani (LB) broth/agar or in M9 minimal growth medium (Darch et al., 2012) supplemented with 0.05% (w/v) phosphatidylcholine (PCh, Sigma).

### Plasmid Construction

The primers used for the construction of recombinant plasmids are summarized in **Supplementary Table 2**. Complete sequences of *rhlR*, *fleQ*, *hcp1*, and *clpV1* genes were amplified from the genomic DNA of PA using primers with the HindIII/KpnI restriction site at their ends followed by double digestion. Then the *rhlR* and *hcp1* genes were ligated into HindIII-KpnI-digested plasmid pAK1900 to generate pAK1900*rhlR* and pAK1900*hcp1*, respectively; *fleQ* and *clpV1* were ligated into HindIII-KpnI-digested pMQ70 to generate pMQ70*fleQ* and pMQ70*clpV1*. Recombinant plasmids were transformed into competent cells of corresponding PA mutant strains for gene complementation by heat-shock or electroporation. The successful complementation of each gene was confirmed by PCR amplification and DNA sequencing.

### Culture Conditions

A proximity assay was performed as previously described with slight modifications (Keogh et al., 2016). Overnight cultures of PA, SA, and KP, which were started from a single colony, were first normalized to 1–2 × 10^8^ CFUs/ml in PBS to OD_600 nm_ (optical density) ≈ 0.1, 0.13, and 0.11 for PA, SA, and KP, respectively. Approximately 1–2 × 10^6^ CFUs of inocula prepared in a total volume of 1 μl were spotted on LB agar with gradient interspecific distances and cultured at 37°C for different time phases. Among the three species studied, only SA is susceptible to ampicillin (Amp) and only KP (with plasmid pRU1103) is resistant to gentamicin (Gm). Macrocolonies were excised and resuspended in PBS, and gradient dilutions were spread on blank LB plates and LB plates containing designated antibiotics. The plates with uniformly distributed colonies and a large spacing between two colonies were selected for CFU enumeration. The number of SA was calculated by the colony number on the blank LB plate minus that on the Amp-LB (100 μg/ml) plate, and then the number of KP was determined by directly counting the colonies on the Gm-LB (15 μg/ml) plate. The number of PA was calculated by the colony number on the blank LB plate minus the total numbers of SA and KP. Moreover, the phenotype differences of PA (flat surface with light green color that turns brown on the following day), SA (small-round-shape with yellow color), and KP (raised and sticky surface with faint yellow color) on the blank LB plate were also used to verify the proportions of target species. The two-species proximity assay between the clinical isolates SWM2 and SWM21 with CA or EC were also performed as described above. The blank LB and Amp-LB (100 μg/ml) plates were used together to calculate their CFUs in the mixture, because only SWM2 and SWM21 are resistant to Amp.

For short-period culture, a total of 1.0 × 10^7^ CFUs of pure PA, SA, and KP, or mixed PA with SA or KP at different ratios (1:9 and 9:1) were inoculated in 4 ml M9-PCh (0.05%) broth with shaking (220 rpm). The population proportions were determined by transferring 50 μl of culture liquids at defined sampling points onto the LB plate and selective media after appropriate dilutions. For the *in vitro* evolution assay, equal total amounts (1.0 × 10^7^ CFUs) of PA, SA, and KP at different combinations were repeatedly cultured in 4 ml M9-PCh broth and the medium was refreshed at 24 h intervals. The population size and proportion of each species were calculated at the end of each cycle, and a total of 50 PA colonies were also transferred onto an M9-caseinate sodium (0.5%, w/v) plate to identify their protease activity (Zhao et al., 2016) and the DNA sequence of the *lasR* gene (**Supplementary Table 2**).

### RNA-Seq and Transcriptomic Analysis

Bacterial cells from different culture conditions were harvested for total RNA isolation using TRIzol reagents (Invitrogen). To minimize the potential deviation of RNA-Seq, RNA samples of three independent experiments were well mixed and sequenced by Novogene Bioinformatics Technology Co., Ltd. (Beijing, China) using prokaryotic strand-specific Illumina-based RNA-Seq technology. Transcriptomic data are deposited at the NCBI database under accession number SRP111420. The software Tophat2 (Kim et al., 2013) was used to map the clean reads to the reference genome of PAO1 (NCBI accession number: AE004091). The software package Cufflinks (Trapnell et al., 2012) was used to obtain transcriptome assembly and calculate the values of differential gene expression using expected fragments per kilobase of transcript per million fragments (FPKM). A differentially expressed gene with a false discovery rate (FDR) < 0.05 was thought to be significantly different. Statistics of gene numbers was performed by using VENNY 2.1^1^. A heat map was generated by using HemI (Deng et al., 2014). KEGG analysis was performed by using KEGG Mapper^2^ (last updated: November 4, 2014) and DAVID Bioinformatics Resources^3^ (Huang et al., 2009).

### Quantitative PCR

Quantitative PCR was performed to validate the results of RNA-Seq by using the QIAGEN OneStep RT-PCR Kit (QIAGEN) per the manufacturer’s instructions. The specific primers used in this study are summarized in **Supplementary Table 2** in the supplemental material. Gene expression was calculated by the 2^-ΔΔCT^ method using 16S rRNA as reference (Zhao et al., 2014).

### Ethics Statement

The sputum samples from the COPD patient were received from an already-existing collection of the respiratory intensive care unit of the affiliated hospital of Southwest Medical University. As the patient passed away the following year, written informed consent to use the samples was obtained from the patient’s immediate family members. The relevant study was approved by Southwest Medical University Ethics Committee, and all methods were carried out in accordance with the guidelines and regulations of Southwest Medical University.

Mice were obtained from the Laboratory Animal Center of Sichuan University (Chengdu, China). The entire animal experimental protocols were performed in strict accordance with good animal practice as defined by the Guide for the Care and Use of Laboratory Animals of the National Institutes of Health (8th edition, 2011), and approved by the guidelines of Institutional Animal Care and Use Committee of Sichuan University (WCCSIRB-D-2016-031).

### Mice Models

Overnight-cultured PA, SA, and KP were harvested and diluted into OD_600_ = 0.5 by sterile saline. C57BL/6J female mice (8-week-old) were anesthetized by intraperitoneal injection of ketamine (50 g/ml) in sterile saline. Equal total amounts (1–2 × 10^6^ CFUs) of agar-bead-encapsulated PA, SA, KP, and 1:1:1 mixture of the three in 50 μl PBS was intranasally instilled into the lung of mice (30 mice per group) as described previously (Facchini et al., 2014). Mice were killed at designated times and the whole lungs were aseptically removed for CFU enumeration. The whole lungs of mice challenged with the mixture of PA, SA, and KP were aseptically removed and about 0.1–0.2 g lung tissue was aseptically excised and homogenized in sterile saline for CFU enumeration. Only the numbers of identified PA, SA, and KP were subjected to proportion calculation.

### Statistical Analyses

Data analysis and statistical tests were performed by using GraphPad Prism version 7.0 (San Diego, CA, United States). Mean values of standard deviation (SD) were compared by using two-tailed unpaired *t*-test or one-way ANOVA with Tukey’s *post hoc* test using a 95% confidence interval.

## Results

### PA Has Higher Growth Advantage When Competing With SA and/or KP

**FIGURE 1:**
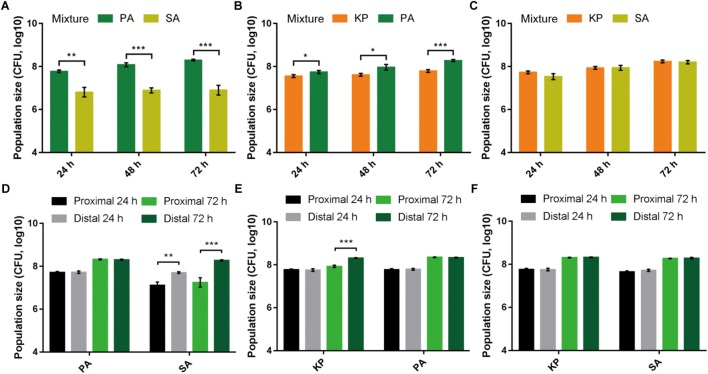
Pairwise interspecific relationship among PA, SA, and KP. **(A–C)** PA can suppress the growth of cocultured SA or KP. Equal amounts (1–2 × 10^6^ CFUs) of PA, SA, and KP were mixed (1:1) in pair and coinoculated on LB plates. **(D–F)** PA proximity can suppress the growth of neighboring SA or KP. Equal amounts (1–2 × 10^6^ CFUs) of PA, SA, and KP were separately inoculated on LB plates in pair and with gradient distances between the two inocula. The population size of each species was determined by CFU enumeration at defined time phases. Data shown are the mean values ± SD of three independent experiments. Statistical significance by two-tailed unpaired *t*-test is indicated: ^∗^*P* < 0.05, ^∗∗^*P* < 0.01, and ^∗∗∗^*P* < 0.001.

To preliminarily understand the relationship of PA with other bacterial species, SA and KP as the commonly isolated G^+^ and G^-^ bacteria from respiratory tract infections were used in this study. Equal amounts of them were mono- or coinoculated on LB plates and cultured for different time phases. The result showed that in comparison to the similar population sizes of PA, SA, and KP in monoculture, PA could significantly suppress the growth of cocultured SA and/or KP (**Figures 1A–C** and **Supplementary Figure 1**). Additionally, as determined by the three-species proximity assay, the population sizes of SA and KP close to PA macrocolonies were significantly decreased compared to that with a greater distance, especially the growth of SA (**Supplementary Figures 2**, **3**). Unexpectedly, a portion of PA was identified from the macrocolonies of KP during prolonged culture (**Supplementary Figures 2C–F**), and this was confirmed by monoclonal phenotypic discrimination and 16S rDNA sequencing. Similarly, when PA, SA, and KP were applied to the two-species proximity assay, PA proximity could significantly inhibit the growth of SA after 24 h, while the population of KP was reduced only when PA emerged in KP macrocolonies, and no significant inhibitory effect was observed between KP and SA (**Figures 1D–F** and **Supplementary Figure 4**). By contrast, the growth of PA was normal and unrelated to the distance from the other two species (**Figures 1D,E**), indicating that the extracellular products of SA and KP had no significant effects on the growth of PA. These results suggested that PA had the highest competitiveness among the three tested species. The inhibitory effect of PA on SA was attributed to the secreted extracellular products of PA, while the growth of KP could be suppressed only when KP was cocultured with PA.

### PA, SA, and KP Can Coexist During Evolution

**FIGURE 2:**
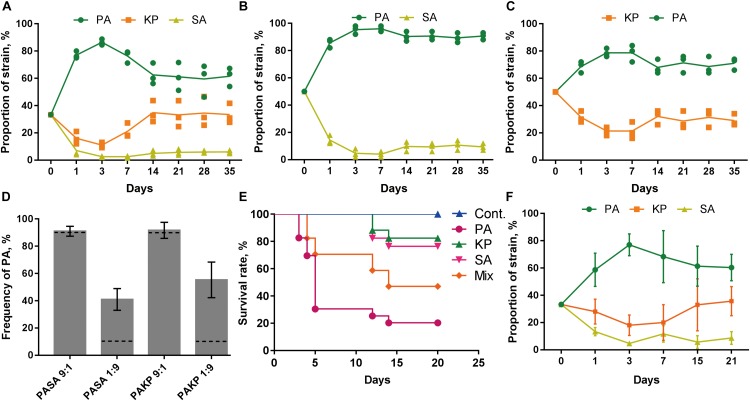
PA is the dominant species during the coevolution with SA and/or KP. **(A–C)** Dynamic proportion changes of PA, SA, and KP when they were repeatedly (24 h intervals) cocultured in different combinations in M9-PCh broth. Cocultures were started from equal species proportion. **(D)** Competition assay. PA, SA, and KP were coinoculated in M9-PCh broth in pair and with different ratios. The final proportion of PA in each coculture was determined after 24 h. **(E)** Lethality of mice intranasally challenged with equal total amounts (1–2 × 10^6^ CFUs) of agar-bead-encapsulated PA, SA, KP, and 1:1:1 mixture of the three in 50 μl PBS (30 mice per group). **(F)** Proportions of coinfected PA, SA, and KP in mouse lungs at different sampling points. Data shown are the means (lines) for the replicates (symbols) **(A–C)** or the mean values ± SD **(D,F)** of three independent experiments.

We then investigated the population dynamics of PA, SA, and KP during evolution by repeatedly (24 h intervals) coculturing them in different combinations. Phosphatidylcholine (PCh), which is the most abundant lung surfactant lipid molecule that can be used for the high cell density proliferation of pathogens in chronic lung infection (Son et al., 2007), was used as the sole nutrient source. The growth rates of monocultured PA, SA, and KP in M9-PCh broth were almost the same in 28 h (**Supplementary Figure 5**). However, the results of the *in vitro* evolution assays showed that PA always had the highest proportion when cocultured with SA and/or KP (**Figures 2A–C** and **Supplementary Figure 6**), and this was consistent with the finding that PA could easily invade the populations of the other two species as determined by the competition assay (**Figure 2D**). Interestingly, although the growths of SA and KP were significantly inhibited in the initial rounds, their proportions were slightly increased during further coculture. Finally, SA and KP could coexist with PA in the pool and form a dynamic equilibrium after 14 cycles. Differently, KP showed a higher degree of proportion increase and fluctuation compared to SA, indicating that in the current experimental condition, SA had the weakest competitiveness among the three species and a more detailed conflict happened between PA and KP during further coexistence (**Figures 2A–C**). Furthermore, when PA, SA, and KP were used to chronically infect the lung of mouse models, mouse deaths were stopped after 15 days and the mice challenged with pure PA showed the highest lethality followed by the mixture of three species (**Figure 2E**). Notably, PA, SA, and KP could also form a relatively stable microcommunity in mouse lungs and showed similar population dynamics with that of *in vitro* coevolution (**Figure 2F**). Our data here revealed that the competitive advantage of PA might be decreased during evolution, thus enabling the coexistence of other species in the PA-dominated microcommunity.

### Population Divergence of PA Contributes to the Coexistence With SA and KP

**FIGURE 3:**
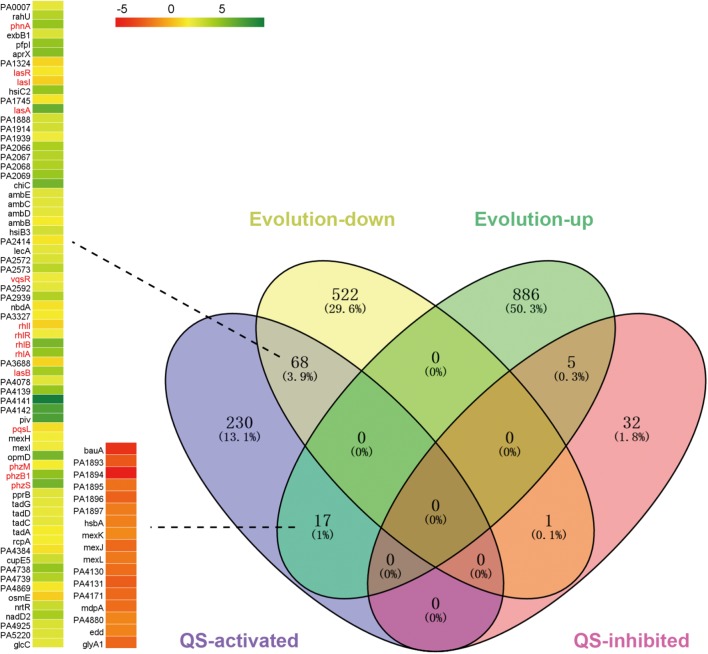
The QS regulation of PA will be decreased during evolution. The global transcriptional profile of repeatedly (24 h intervals) cultured PA in M9-PCh broth at day 21 was compared to that of day 1. The significantly differentially expressed genes in the evolved PA were applied to the regulatory profile of QS system (Δ*lasR-rhlR* vs. wild type) published by Schuster et al. (2003) using VENNY 2.1. Data shown are FDR < 0.05.

To further explore why the competitive advantage of PA was decreased during evolution and how PA could coexist with SA/KP in the microcommunity, the transcriptional changes of PA during evolution in M9-PCh broth were determined by RNA-Seq. When the global transcriptional profile of repeatedly monocultured PA at day 21 (according to the maintenance of two- or three-species dynamic equilibrium) was compared to that of day 1, a total of 908 upregulated and 591 downregulated genes were detected (**Figure 3**). Because previous studies suggested that the QS system of PA was implicated in interspecific interaction (Korgaonkar et al., 2013; Frydenlund Michelsen et al., 2016; Hotterbeekx et al., 2017), our current study mainly focused to explore the performance of the PA QS system during evolution. The significantly differentially expressed genes were then mapped to the regulatory profile of the PA QS system (Δ*lasR-rhlR* vs. WT) published by Schuster et al. (2003) to generate a list of genes under QS control. Among the 315 QS-activated genes, 68 genes including *lasA*, *lasB*, *rhlA*, and *rhlB* as well as the central regulators *lasR* and *rhlR* (1.7 and 2.2-fold decline) were all significantly downregulated at day 21 (**Figure 3**, **Supplementary Figure 7** and **Supplementary Dataset 1**). Additionally, various *lasR* mutants of PA were detected from *in vitro* evolution of pure PA and coevolution of PA with SA and/or KP (**Supplementary Table 3**). These findings suggested that the QS regulation of PA was decreased during evolution by selecting the individuals with a deficient QS system. We then speculated that the decreased competitiveness of PA and the development of interspecific coexistence might be related to the QS-directed population divergence. As expected, the *lasR* mutant (PA-Δ*lasR*) or *rhlR* mutant (PA-Δ*rhlR*) of PA failed to reduce the population sizes of cocultured SA/KP in comparison to the inhibitory effect of WT PA, and similar results were also observed when the clinical PA isolates with an intact- or deficient-QS system were cultured with other coisolated G^-^ and G^+^ species from a COPD patient (**Supplementary Figures 8**–**11**). Therefore, these results collectively demonstrated that the decreased QS regulation of PA could contribute to the formation of a PA-dominated microcommunity.

### Comparative Transcriptomic Analyses of *in vitro* Coevolution

**FIGURE 4:**
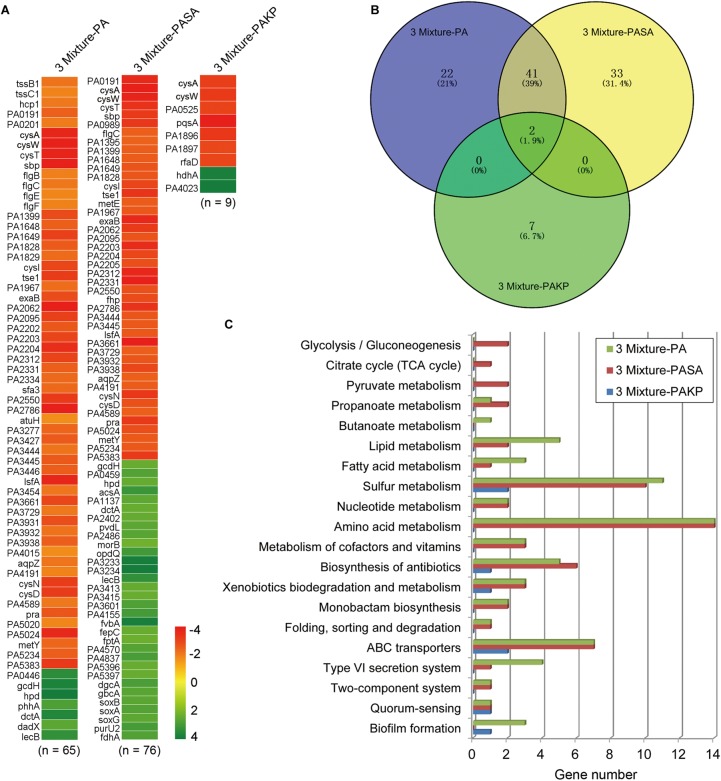
The intracellular transcriptional profile of PA can be influenced by the presence of KP. Different combinations of PA, SA, and KP were repeatedly (24 h intervals) cocultured in M9-PCh broth. Total RNAs of each culture were isolated at day 21. **(A)** Significantly differentially expressed genes of PA when PA was cultured in different conditions. **(B)** Statistics of the numbers and **(C)** KEGG analyses of significantly differentially expressed genes of PA when PA was cultured in different conditions [according to **(A)** and **Supplementary Datasets 2**–**4**] by using VENNY 2.1 and KEGG Mapper. 3 Mixture-PA, coevolution of PA, SA, and KP compared to monoevolution of PA; 3 Mixture-PASA, coevolution of PA, SA, and KP compared to coevolution of PA and SA; 3 Mixture-PAKP, coevolution of PA, SA, and KP compared to coevolution of PA and KP. “*n*” indicates the number of genes. Data shown are FDR < 0.05.

**FIGURE 5:**
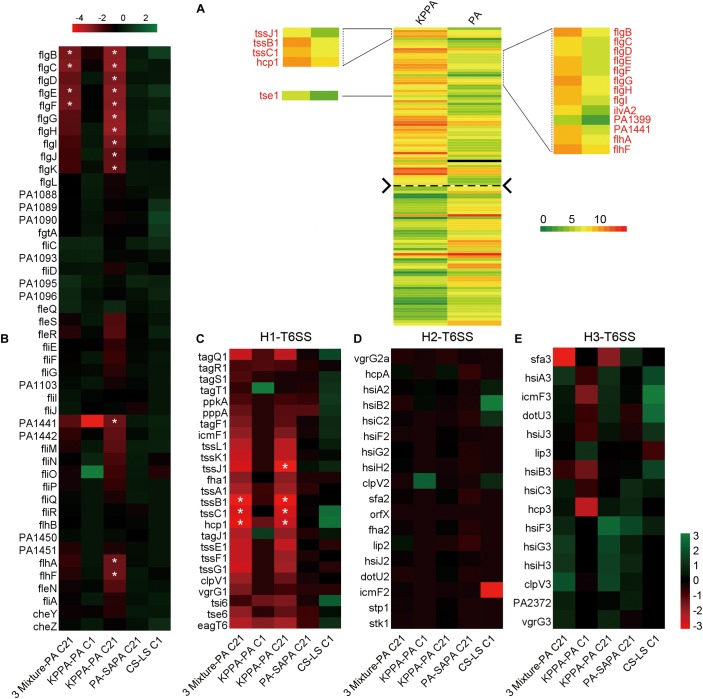
The flagella- and H1-T6SS-related genes of PA are significantly differentially expressed by the presence of KP during evolution. **(A)** Comparison of the global transcriptional profiles of PA between monoevolution of PA and PA-KP coevolution. Total RNAs of each culture were isolated at cycle 21 (day 21). The up- and downregulated genes were separated by the dashed line. Differential expression of **(B)** flagella-related genes, **(C)** H1-, **(D)** H2-, and **(E)** H3-T6SS related genes of PA when PA was cultured in different conditions. 3 Mixture-PA C21, coevolution of PA, SA, and KP compared with monoevolution of PA (cycle 21); KPPA-PA C1, coculture of KP and PA compared with monoculture of PA (cycle 1); KPPA-PA C21, coevolution of KP and PA compared with monoevolution of PA (cycle 21); PA-SAPA C21, monoevolution of PA compared with coevolution of SA and PA (cycle 21); CS-LS C1, monoculture of PA in M9-casamino acids broth at the 24 h time point compared to that in M9-PCh broth. ^∗^FDR < 0.05.

After knowing that the QS ability of PA would be decreased during evolution, we then set out to understand the performances of PA in the initial competition and in stabilizing the coexistence with SA and KP by using comparative transcriptomic analysis. No typical QS-related genes of PA were enriched when the transcriptional profile of PA from three-species coevolution (day 21) was compared to that of monoevolution (**Figures 3**, **4A** and **Supplementary Dataset 2**). This suggested that the QS regulation of PA was also decreased in stabilizing the three-species coexistence. Interestingly, the global transcriptional pattern of PA during the maintenance of PA-dominated three-species equilibrium was more similar to that of PA-KP coevolution (**Figures 4A,B** and **Supplementary Datasets 2**–**4**). KEGG analysis showed that the incorporation of KP into the microcommunity could increase the lipid metabolism, sulfur metabolism, biosynthesis of antibiotics, ABC transporters, and the T6SS of PA, but decrease the expression of amino acid metabolism-related genes (**Figure 4C** and **Supplementary Figure 12**). By contrast, the presence of SA had less effect on the intracellular expression of PA. These results were consistent with the population dynamics of PA, SA, and KP during coevolution (**Figures 2A,F**), and indicated that PA might have more frequent interactions with KP irrespective of the existence of SA. This could be supported by our further results that no significantly differentially expressed gene was detected when the transcriptional profile of monocultured PA at day 21 was compared to that of PA-SA coevolution (**Supplementary Dataset 5**), while 61 upregulated genes and 54 downregulated genes of PA were detected when PA was repeatedly cocultured with KP for 21 days (**Figure 5A** and **Supplementary Dataset 6**). These findings, combined with the remarkably low proportion of SA and the fluctuant proportion of KP during two- or three-species coevolution (**Figure 2**), confirmed that in the PA-dominated microcommunity of our current experimental system, the decreased QS regulation of PA allowed the persistent coexistence of SA and KP, and the interspecific competition among the three tested species mainly happened between PA and KP during further evolution.

### Enhanced H1-T6SS of PA Can Restrict the Growth of KP

**FIGURE 6:**
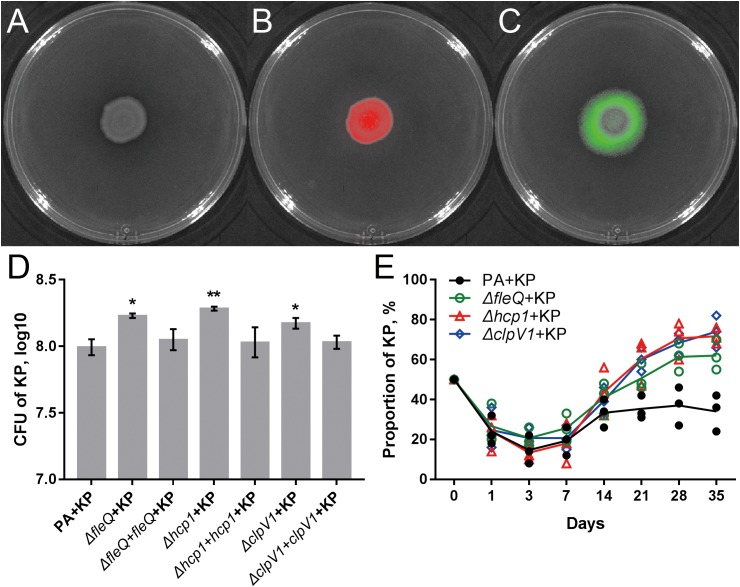
Flagella and H1-T6SS of PA contribute to the coexistence of PA with KP. **(A–C)** Coculture of 1:1 mixed PA-mCherry and KP-GFP on LB plate for 4 days. Red color shows PA-mCherry, and green color shows KP-GFP. **(D)** Population sizes of KP when KP was mixed (1:1) and coinoculated with different strains of PA on LB plate at the 48 h time point. **(E)** Dynamic proportion changes of KP when KP was repeatedly cocultured with different strains of PA at 24 h intervals in M9-PCh broth. Data shown are the **(D)** mean values ± SD, or **(E)** means (lines) for the replicates (symbols) of three independent experiments. Statistical significance by one-way ANOVA with Tukey’s *post hoc* test using a 95% confidence interval (compared to the value of PA + KP) is indicated: ^∗^*P* < 0.05, ^∗∗^*P* < 0.01.

The result of comparative transcriptomic analysis revealed two remarkable categories of PA genes, the flagella- and H1-T6SS-encoding genes, that were significantly upregulated when PA was repeatedly cocultured with KP for 21 days compared to that of monocultured PA (**Figure 5A**, **Supplementary Dataset 6** and **Supplementary Figure 13**). Importantly, these two gene clusters of PA were upregulated only when KP was included in the evolved microcommunity but unrelated to the presence of SA (**Figure 5B**). Moreover, the expression levels of H2- and H3-T6SS-related genes of PA showed no significant change. We also checked the performance of PA flagella and H1-T6SS when PA was cocultured with KP for a short period in M9-PCh or in M9-casamino acids (CAA) by using RNA-Seq. Unexpectedly, no significant differential expression of any relevant genes were detected, and the majority of these genes were not even expressed in these conditions (**Figures 5B–E** and **Supplementary Datasets 7**–**9**). In comparison to the rapidly upregulated H1-T6SS of PA when PA was competing with other G^-^ bacteria on solid medium (LeRoux et al., 2015), our data here suggested that the H1-T6SS of PA would be activated during the development of PA-dominated microcommunity in liquid culture, and this might be due to the autoaggregation of bacterial cells (sink to the bottom) that could also create a condition of direct cell–cell contact. To further investigate the role of flagella and H1-T6SS of PA in restricting the growth of KP, the mCherry-labeled PA and GFP-labeled KP were well-mixed and coinoculated on the LB plate. Interestingly, KP was mainly distributed in the peripheral region of the cocultured macrocolony compared to the uniformly distributed PA (**Figures 6A–C**), and the expression of the PA *hcp1* gene in the inner region was significantly higher than that of the outer (**Supplementary Figure 14**). We then performed a proximity assay by using KP and the motionless strain of PA (PA-Δ*fleQ*) (Arora et al., 1997); the result showed that in comparison to the directionally trans-missed WT PA to KP on the LB plate, the surface of KP macrocolonies remained tidy when KP was close to PA-Δ*fleQ*, while the transmission was reproduced when the *fleQ* gene was restocked to PA-Δ*fleQ* (**Supplementary Figure 15**). Furthermore, when PA-Δ*fleQ* or H1-T6SS mutants (PA-Δ*hcp1* and PA-Δ*clpV1*) were coinoculated with the KP on LB plate or repeatedly cocultured in M9-PCh broth, all these mutant strains of PA failed to restrict the proportion increase of KP during further evolution (**Figures 6D,E**). Taken together, our findings uncovered that the decreased QS regulation and the enhanced H1-T6SS of PA could jointly lead to the dynamic coexistence of KP with PA during the maintenance of a relatively stable microcommunity.

## Discussion

As a common inhabitant of the human respiratory tract, PA frequently shares the same ecological niche with other pathogens in the lung environment and this coexistence model can synergistically cause the chronic symptoms (Short et al., 2014; Wunderink and Waterer, 2014). Understanding the strategy of PA in interspecific interactions has important biological and clinical implications. In this study, we show that although the development of the QS system endows PA a significant growth advantage and enables the success of PA in invading the niches initially dominated by other pathogens, the subsequent QS-mediated population divergence and the enhanced T6SS-related cell aggressivity of PA can jointly facilitate the coexistence of PA with other species during evolution.

We first study the general relationships of PA with SA and/or KP by using proximity and coevolution assays. PA is confirmed to be the dominant species, and these three pathogens can form a relatively stable microcommunity during *in vitro* evolution and in causing chronic lung infection (**Figures 1**, **2**). Previous studies have clearly confirmed that the QS-controlled extracellular products of PA can suppress the growth of cocultured SA *in vitro* and *in vivo* (Mashburn et al., 2005; Korgaonkar et al., 2013; Hotterbeekx et al., 2017). These are according to the natural development of cystic fibrosis lung disease that is colonized by SA initially, and the subsequently invaded PA will become the most significant pathogen (Kahl, 2010; Bevivino and Bragonzi, 2013; Yonker et al., 2015; Maliniak et al., 2016). Frydenlund Michelsen et al. (2016) identified that a clinical strain DK2-P2M24-2003 with deficient QS regulation could form a mutualistic symbiotic relationship with neighboring SA by synthesizing a distinct 4-hydroxy-2-alkylquinoline profile upon the presence of SA with a functional *agr* system. Notably, the DK2 lineages of PA with reduced production of virulence factors are supposed to be highly successful and transmissible (Yang et al., 2011; Feliziani et al., 2014). Therefore, the decreased QS-related competitiveness of PA can contribute to the coexistence of other species in the PA-dominated microcommunity. This hypothesis can be well supported by our finding that although the growth of SA is significantly suppressed by PA initially, the subsequent population divergence of PA caused by the enrichment of *lasR* mutants creates a condition for the persistent survival of SA during further evolution. LasR is the central regulator of the PA QS hierarchy, and various *lasR* mutants can be frequently detected from a series of *in vitro* evolution assays due to the increased social conflict for resources (Heurlier et al., 2005; Sandoz et al., 2007; Eldar, 2011; Dandekar et al., 2012; Zhao et al., 2016). During the coevolution of PA and SA, PA as the absolute dominant species mainly competes with its homogeneous individuals rather than with SA for space and resources (**Figure 2B** and **Supplementary Dataset 5**). In this trend, the emergence of PA *lasR* mutants can simultaneously relieve the QS-imposed metabolic burden of PA in producing the costly extracellular products (Smith and Chapman, 2010; Eldar, 2011; Zhao et al., 2016) and the survival stress of SA during further coevolution (**Figure 3** and **Supplementary Figure 8**).

Unlike the inhibitory effect of PA on G^+^ pathogens through quorum-controlled extracellular products, the interactions of PA with other G^-^ bacteria are much more complicated. In our current experimental system, PA has more frequent interactions with KP in the microcommunity as confirmed by the coevolution assays and comparative transcriptomic analyses (**Figures 2**, **4**). However, due to the limitation of the detailed intracellular regulatory information on KP (especially the potential QS system), there are only a few studies reporting the interspecific interaction between PA and KP. Kelvin Lee et al. (2016) found that coculture of PA and KP could reduce the intraspecific diversity of each species but enhance the tolerance of the mixed population against sodium dodecyl sulfate stress, and these processes might be attributed to the effect of each other’s extracellular factors. Here we show that PA has a higher competitive advantage than KP when they are cocultured in a tube or on a solid medium (**Figures 1B**, **2C**). It seems that the suppressed growth of KP is unrelated to the toxicity of extracellular products from PA, because PA proximity has no significant effect on the growth of KP before the presence of PA on KP macrocolony (**Figure 1E** and **Supplementary Figures 4C,D**). However, the growth of KP is comparable to that of cocultured PA QS mutants and few of PA can be detected on KP macrocolonies in the proximity assay (**Supplementary Figures 9**, **10**). These findings suggest that the development of the QS system can enhance the advantage of PA when competing with KP. Although we have shown that the knockout of the *fleQ* gene can block the transmission of PA toward KP (**Supplementary Figure 15**), it is hard to explain why PA only emerges on the surface of KP macrocolonies instead of SA and without leaving any traces (**Supplementary Figure 4**). Our work here primarily explores the performance of PA against KP, and the metabolite-mediated interspecific communication and the underlying mechanism will be further investigated along with the detailed characterization of intracellular regulation and extracellular products of KP.

In comparison to the significantly compressed population size of SA in PA-dominated microcommunity, the decreased QS regulation and the enhanced H1-T6SS of PA can cause a remarkable population fluctuation of KP during further coevolution (**Figures 2**, **4**, **5**). It is reported that the RetS mutant of PA produces a high level of c-di-GMP and can upregulate the H1-T6SS through Gac/Rsm signaling (Goodman et al., 2004; Moscoso et al., 2011), and LasR that sits at the bottom of Gac/Rsm cascades can negatively regulate the H1-T6SS (Schuster et al., 2003; Lesic et al., 2009). In this point, it is reasonable that the population of PA with decreased QS regulation can still restrict the prevalence of KP by activating the H1-T6SS. Moreover, c-di-GMP can inhibit the cell mobility of PA by competitively inhibiting the effect of FleQ (Ventre et al., 2006; Baraquet et al., 2012), while the FleQ mutant displays decreased intracellular c-di-GMP levels and indirectly weakens the expression of T6SS (Hickman and Harwood, 2008; Moscoso et al., 2011). Therefore, the activation of T6SS in PA is much more complicated and can be multiply controlled by c-di-GMP, the QS system, and the global regulators RetS/GacS. This is supposed to provide a plausible explanation for the prosperity of PA in complex niches shared by various bacterial species. LeRoux et al. (2015) introduced a program termed *P. aeruginosa* response to antagonism and showed that the H1-T6SS of PA could be activated by the cell lysis of kin species. The interspecific interaction between PA and KP identified in our study is in good agreement with this program, which proposes that the activation of the H1-T6SS is related to the competitiveness of PA. Specifically, the H1-T6SS of PA remains inactive when PA can easily overcome KP by using a robust QS regulation. Along with the occurrence of QS-directed social divergence that decreases the competitiveness of PA, the H1-T6SS will be activated to restrict the proportion of KP during further coevolution.

In conclusion, our findings provide a general explanation for the coexistence of *P. aeruginosa* with other bacterial pathogens during evolution and contribute to the emerging interest in multispecies interactions in a local microcommunity. Further studies concerning the discovery of specific *P. aeruginosa* isolates with different auxotrophic phenotypes, antibiotic resistances, or mutations may contribute to identifying more interesting sufficient conditions (such as metabolic cross-feeding, antibiotic-mediated interspecific cooperation, or mutation-caused extracellular product communication) for the formation of microcommunities composed of more microbial species.

## Data Availability Statement

The datasets generated for this study can be found in the NCBI database (https://www.ncbi.nlm.nih.gov/sra/SRP111420).

## Author Contributions

KZ and YC designed the work. KZ, JL, YY, XRW, XWW, and YG performed the experiments. KZ, LD, and BY performed the bioinformatics analyses. YZ coordinated the clinical samples collection and preparation. KZ, YZ, and YC wrote the manuscript.

## Conflict of Interest Statement

The authors declare that the research was conducted in the absence of any commercial or financial relationships that could be construed as a potential conflict of interest.

## Footnotes

1 http://bioinfogp.cnb.csic.es/tools/venny/

2 http://www.kegg.jp/kegg/tool/map_pathway.html

3 https://david.ncifcrf.gov/tools.jsp
