# Supplementary material for: Pseudomonas aeruginosa Quorum-Sensing and Type VI Secretion System Can Direct Interspecific Coexistence During Evolution
Source: Front Microbiol. 2018 Oct 11;9:2287. doi: 10.3389/fmicb.2018.02287 (PMC6193480; doi:10.3389/fmicb.2018.02287)
Supplement: Supplementary file 10 [file Data_Sheet_1.PDF]

Supplementary Material

Pseudomonas aeruginosa Quorum-sensing and Type VI Secretion System Can Direct Interspecific Coexistence during Evolution

Kelei Zhao[1,*], Lianming Du[2], Jiafu Lin[1], Yang Yuan[1], Xiwei Wang[1], Bisong Yue[3], Xinrong Wang[1], Yidong Guo[1], Yiwen Chu[1,*] and Yingshun Zhou[4,*]

[1] Antibiotics Research and Re-evaluation Key Laboratory of Sichuan Province, Sichuan Industrial Institute of Antibiotics, Chengdu University, Chengdu, China

[2] Institute for Advanced Study, Chengdu University, Chengdu, China

[3] Key Laboratory of Bio-resources and Eco-environment (Ministry of Education), College of Life Sciences, Sichuan University, Chengdu, China

[4] Department of Pathogenic Biology, College of Preclinical Medicine, Southwest Medical University, Luzhou, China

*** Correspondence:**
Kelei Zhao, Email: zkl5228@163.com; Yiwen Chu, Email: chuyiwen@cdu.edu.cn; and Yingshun Zhou, Email: yingshunzhou@swmu.edu.cn

Supplementary Figures

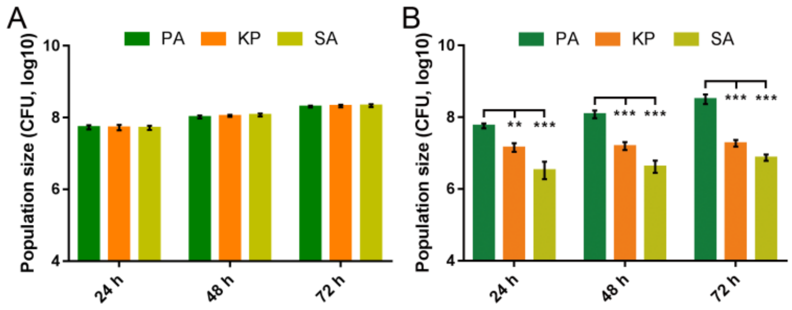

Supplementary Figure 1. PA can suppress the growth of cultured KP and SA. **(A)** Population sizes of mono-cultured PA, KP, and SA on LB plates at defined time-phases. **(B)** Population sizes of PA, SA, and KP when they were mixed (1:1:1) and co-inoculated on LB plates for defined time-phases. Data shown are the mean values ± SD of three independent experiments. Statistical significance was performed by One-way ANOVA with Tukey's post hoc using a 95% confidence interval (compared to the value of PA): $**P < 0.01$, $***P < 0.001$.

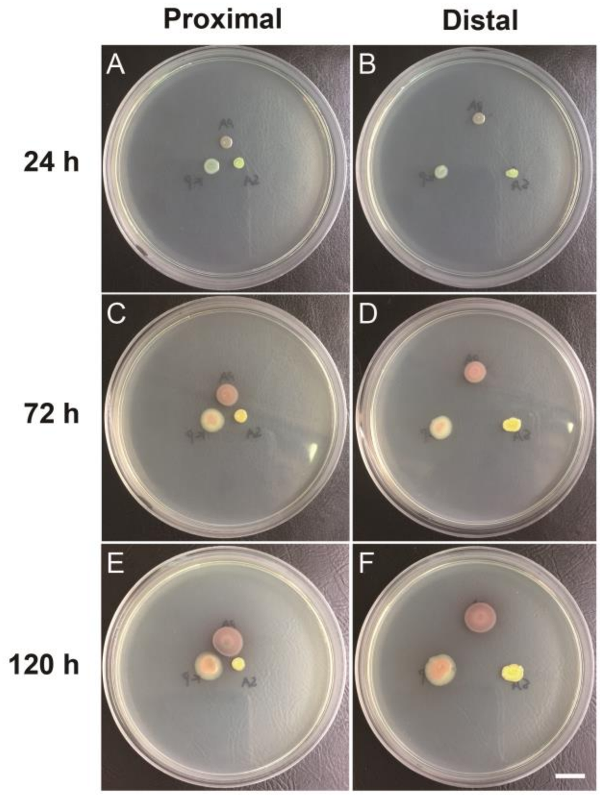

Supplementary Figure 2. Three-species proximity assay of PA, SA, and KP on LB plates. Top, PA. Bottom left, KP. Bottom right, SA. Scale bar, 1 cm.

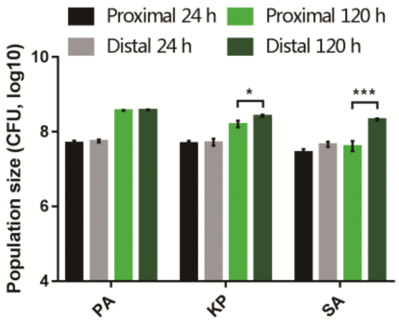

Supplementary Figure 3. Population sizes of PA, SA, and KP in three-species proximity assay. Data shown are the mean values ± SD of three independent experiments. Statistical significance by two-tailed unpaired *t*-test is indicated: $*P < 0.05$, $***P < 0.001$.

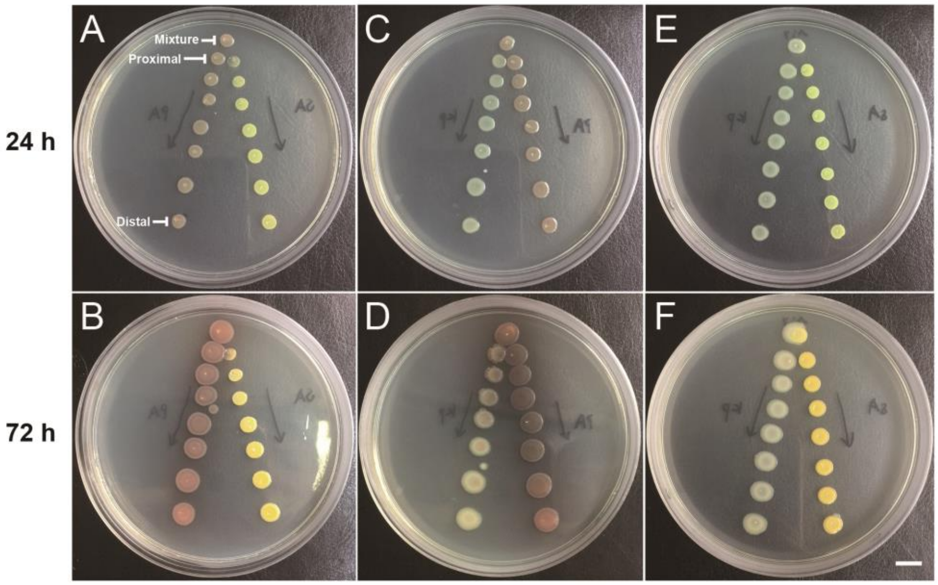

Supplementary Figure 4. Pairwise proximity assay of PA, SA, and KP on LB plates. In panels **(A)** and **(B)**: top, mixture (1:1) of PA and SA; left, PA; right, SA. In panels **(C)** and **(D)**: top, mixture (1:1) of PA and KP; left, KP; right, PA. In panels **(E)** and **(F)**: top, mixture (1:1) of KP and SA; left, KP; right, SA. Scale bar, 1 cm.

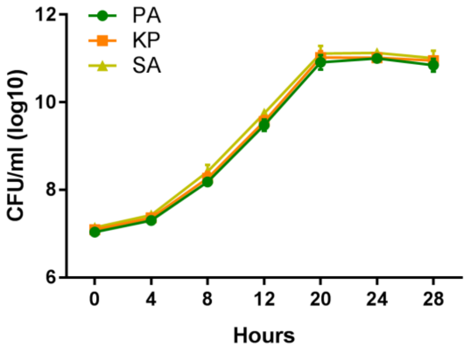

Supplementary Figure 5. Growth curves of PA, SA, and KP when they were separately cultured in M9-phosphatidylcholine (PCh, 0.05%) broth. Data shown are the mean values ± SD of three independent experiments.

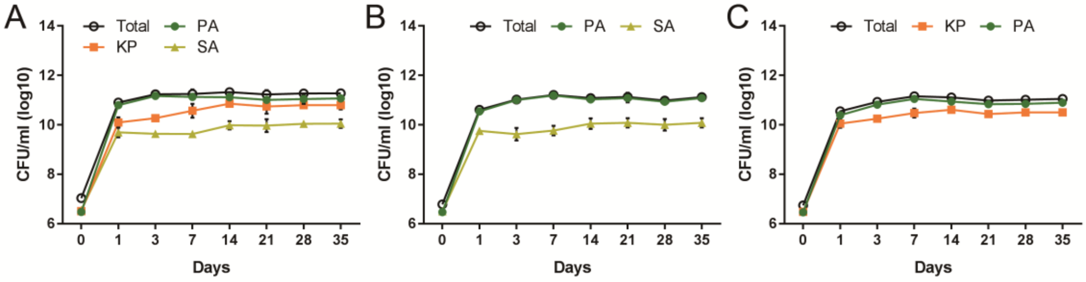

Supplementary Figure 6. Growth curves of PA, SA, and KP when they were repeatedly co-cultured in M9-PCh broth at 24-h intervals. Data shown are the mean values ±SD of three independent experiments.

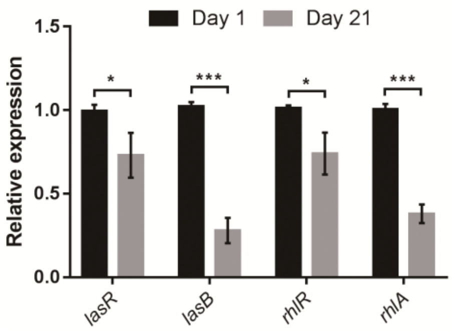

Supplementary Figure 7. Expression of typical QS-related genes of evolved PA as determined by quantitative PCR. Day 1, culture of PA in M9-PCh broth for 24 hours. Day 21, repeatedly culture of PA in M9-PCh broth for 21 days. Data shown are the mean values ± SD of three independent experiments. Statistical significance by two-tailed unpaired *t*-test is indicated: *$P < 0.05$, ***$P<0.001$.

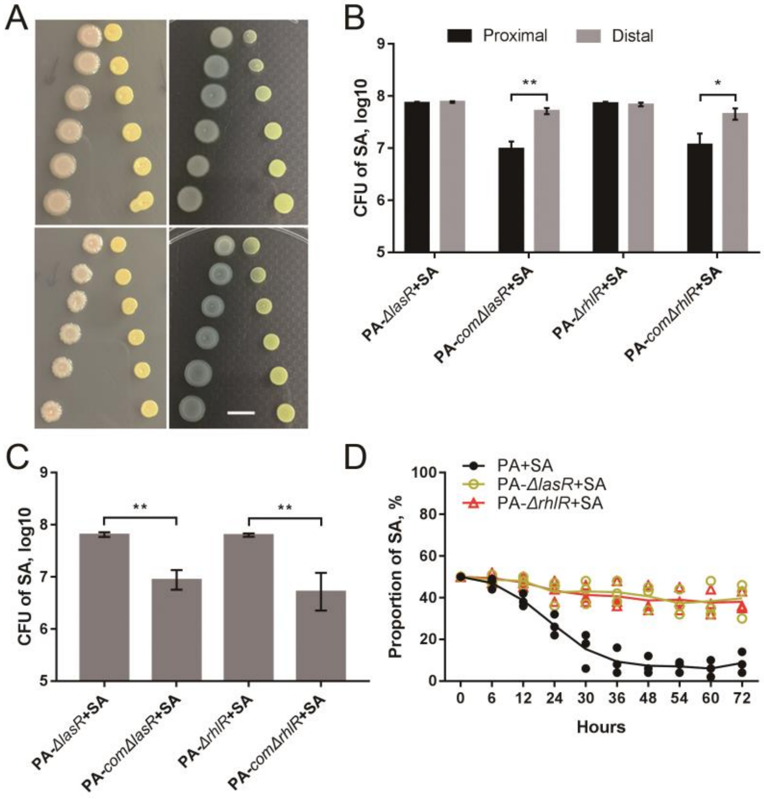

Supplementary Figure 8. PA suppresses the growth of SA by using QS-mediated regulation. **(A)** Proximity assay of PA QS-deficient strains and SA at 48-h time-point. Top left panel: left, PA-*ΔlasR*; right, SA. Top right panel: left, PA-*comΔlasR*; right, SA. Bottom left panel: left, PA-*ΔrhlR*; right, SA. Bottom right panel: left, PA-*comΔrhlR*; right, SA. **(B)** Population size of SA in the proximity assay. **(C)** Population size of SA when SA was mixed (1:1) and co-inoculated with different strains of PA on LB plate at 48 h time point. **(D)** *In vitro* co-culture of SA with different strains of PA. Data shown are the **(B** and **C)** mean values ± SD, or **(D)** means (lines) for the replicates (symbols) of three independent experiments. Statistical significance by two-tailed unpaired *t*-test is indicated: **P* < 0.05, ***P* < 0.01. Scale bar, 1 cm.

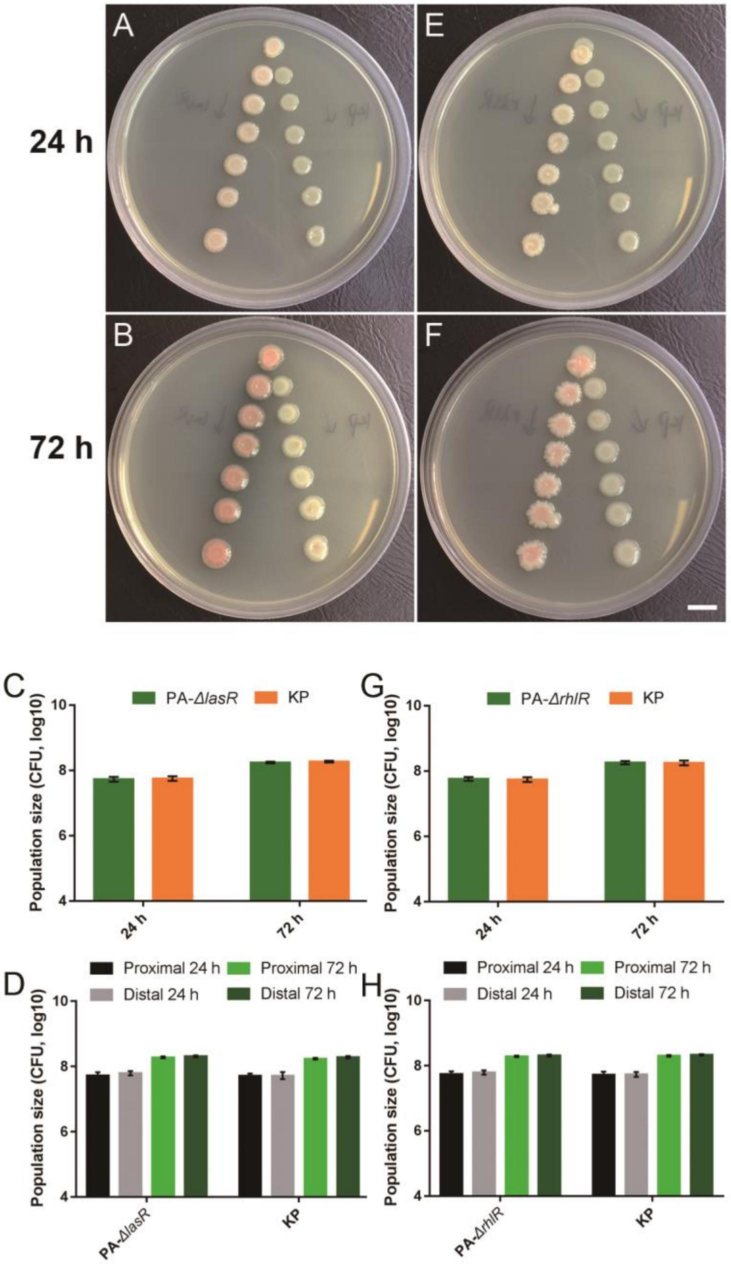

Supplementary Figure 9. QS-deficient mutants of PA have no inhibitory effect on the growth of KP on LB plates. **(A** and **B)** Proximity assay of PA-*ΔlasR* and KP. Left, PA-*ΔlasR*; right, KP. **(C)** Population sizes of PA-*ΔlasR* and KP when they were mixed (1:1) and co-inoculated on LB plate. **(D)** Population size of PA-*ΔlasR* and KP in the proximity assay. **(E** and **F)** Proximity assay of PA-*ΔrhlR* and KP. Left, PA-*ΔrhlR*; right, KP. **(G)** Population sizes of PA-*ΔrhlR* and KP when mixed (1:1) and co-inoculated on LB plate. **(H)** Population size of PA-*ΔrhlR* and KP in the proximity assay. Data shown are mean values ± SD of three independent experiments. Statistical significance was performed by two-tailed unpaired *t*-test. Scale bar, 1 cm.

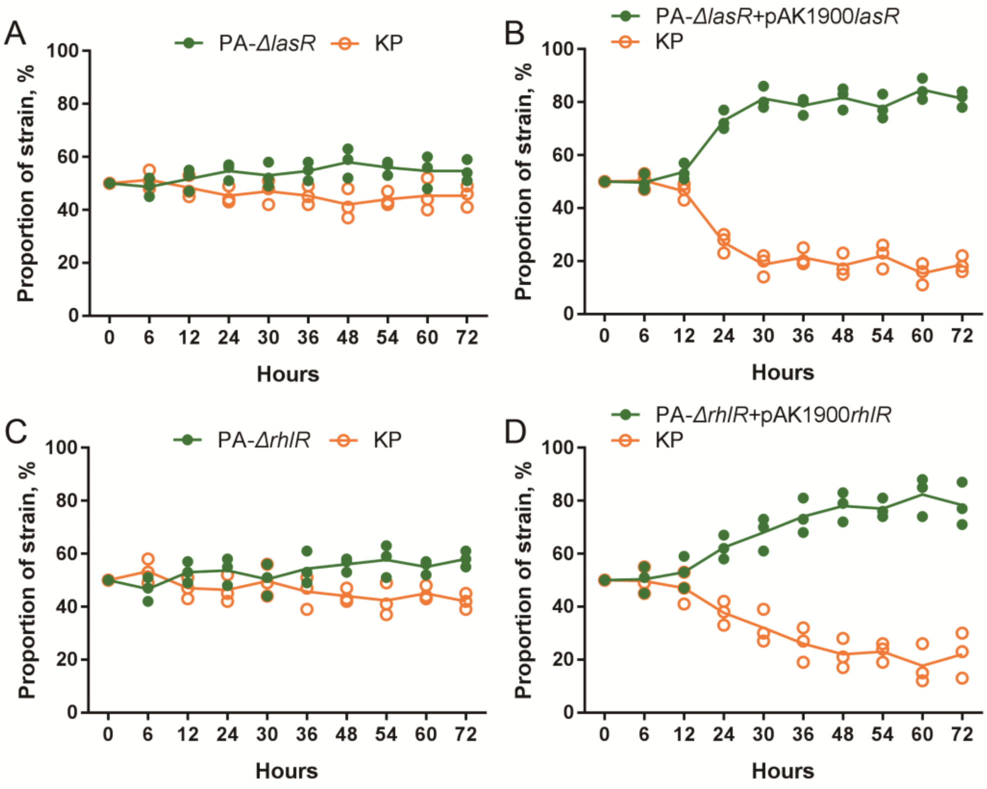

Supplementary Figure 10. QS-mediated regulation of PA can suppress the growth of KP. QS-deficient strains (PA-*ΔlasR* and PA-*ΔrhlR*) and corresponding gene complemented strains (*ΔlasR+lasR* and *ΔrhlR+rhlR*) were co-cultured with KP in M9-PCh, respectively. Co-cultures were started from equal proportion and the subsequent proportions were determined at defined time phases. Data shown are the means (lines) for the replicates (symbols) of three independent experiments.

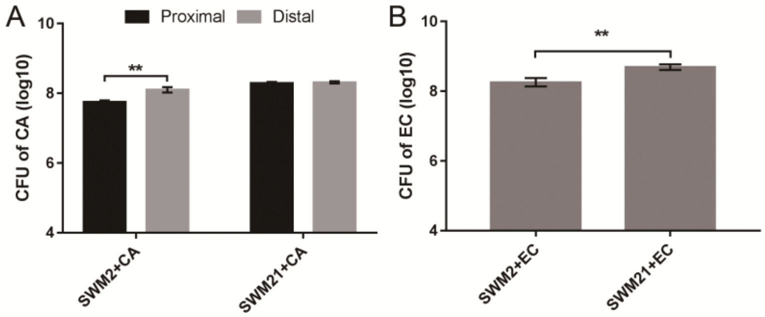

Supplementary Figure 11. Clinical PA with a functional QS system can suppress the growth of co-isolated *Corynebacterium argentoratense* (CA) and *Escherichia coli* (EC). **(A)** Proximity of clinical PA isolate SWM2 can significantly reduce the population of G^+ bacteria CA in comparison to the isolate SWM21 with mutation in *lasR* gene. **(B)** SWM2 can significantly suppress the growth of co-cultured G^- bacteria EC in comparison to SWM21. Data shown are mean values ± SD of three independent experiments. Statistical significance by two-tailed unpaired *t*-test is indicated: $**P < 0.01$.

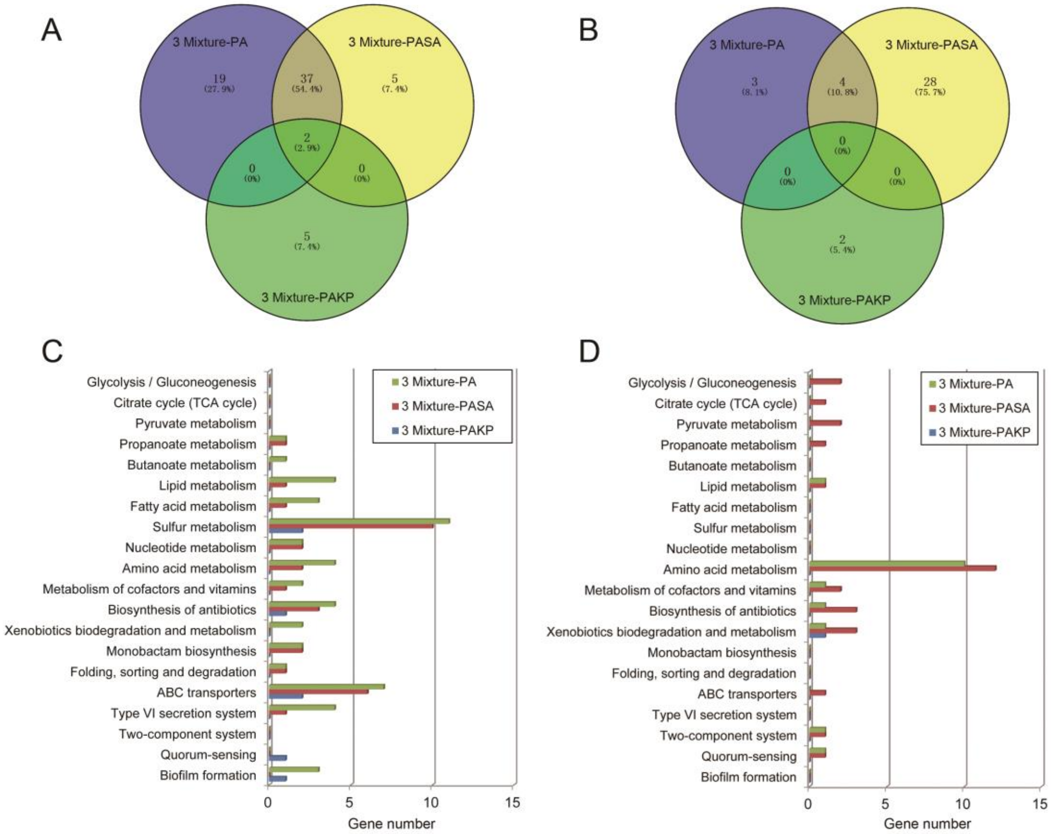

Supplementary Figure 12. Comparative analysis of the differentially expressed genes of PA according to **Figure 4.** Statistics of **(A)** up-regulated and **(B)** down-regulated gene numbers of PA in different experiments was determined by using the online software VENNY 2.1. KEGG analysis of **(C)** up-regulated and **(D)** down-regulated genes of PA in different experiments was performed by using KEGG Mapper. 3 Mixture-PA, co-evolution of PA, SA, and KP compared to mono-evolution of PA; 3 Mixture-PASA, co-evolution of PA, SA, and KP compared to co-evolution of PA and SA; 3 Mixture-PAKP, co-evolution of PA, SA, and KP compared to co-evolution of PA and KP.

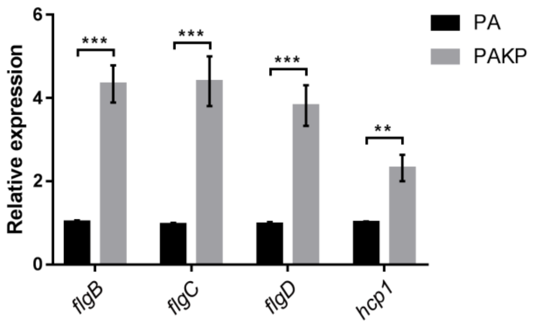

Supplementary Figure 13. Expression of flagella- and H1-T6SS-related genes as determined by quantitative PCR. PA, repeated mono-culture of PA for 21 days. KPPA, repeated co-culture of PA and KP for 21 days. Data shown are the mean values ± SD of three independent experiments. Statistical significance by two-tailed unpaired *t*-test is indicated: $**P < 0.01$, $***P<0.001$.

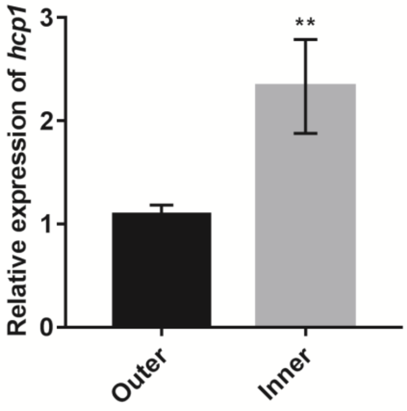

Supplementary Figure 14. Expression of *hcp1* gene of PA in the inner and outer regions of mixed PA-KP macrocolony (according to **Figure 6A–C**) as determined by quantitative PCR. Data shown are the mean values ± SD of three independent experiments. Statistical significance by two-tailed unpaired *t*-test is indicated: $^{**}P < 0.01$.

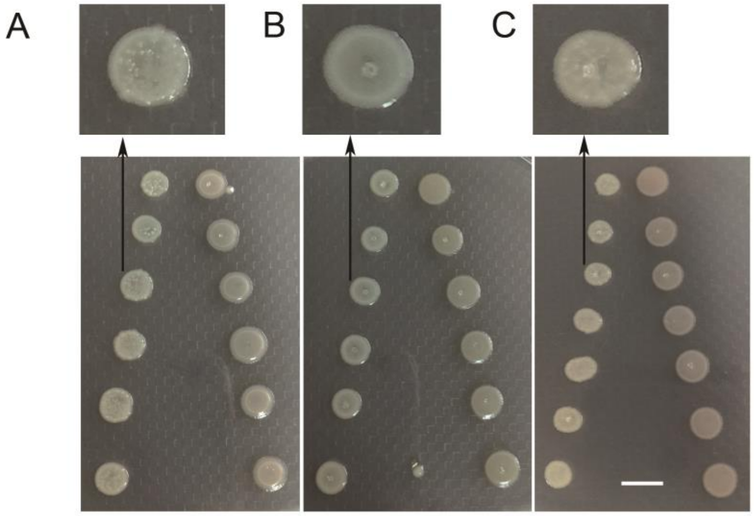

Supplementary Figure 15. FleQ dominates the directional transmission of PA to neighboring KP (48 h). **(A)** Left, KP; right, wild type PA. **(B)** Left, KP; right, PA-*ΔfleQ*. **(C)** Left, KP; right, PA-*comΔfleQ*. Black arrow indicates the presence and absence of PA on the surface of KP macrocolonies. Scale bar, 1 cm.

Supplementary Tables

Supplementary Table 1. Strains and plasmids used in this study.

Strains or plasmids	Description	Reference
Plasmids		
pRU1103	Gm^R, broad-host-range vector for G^- bacteria	Karunakaran et al., 2005
pAK1900	Amp^R, shuttle vector	Deng et al., 2013
pMQ70	Amp^R, shuttle vector	Shanks et al., 2006
pAK1900*lasR*	pAK1900 plasmid with *lasR*	Deng et al., 2013
pAK1900*rhlR*	pAK1900 plasmid with *rhlR*	This study
pMQ70*fleQ*	pMQ70 plasmid with *fleQ*	This study
pAK1900*hcp1*	pAK1900 plasmid with *hcp1*	This study
pMQ70*clpV1*	pMQ70 plasmid with *clpV1*	This study
Strains		
P. aeruginosa		
PA	Wild type PAO1	Zhao et al., 2014
PA-mCherry	PAO1 harbors pMMR express mCherry	Popat et al., 2012
SWM2	Wild type PA from COPD patient	This study
SWM21	LasR mutant PA from COPD patient	This study
ΔlasR	Clean deletion of *lasR* in PAO1	Zhao et al., 2014
comΔlasR	PAO1-*ΔlasR* carrying plasmid pAK1900*lasR*	Deng et al., 2013
ΔrhlR	Clean deletion of *rhlR* in PAO1	Wilder et al., 2011
comΔrhlR	PAO1-*ΔrhlR* carrying plasmid pAK1900*rhlR*	This study
ΔfleQ	PAO1 containing a transposon insertion	Jacobs et al., 2003
ΔfleQ+fleQ	PAO1-*ΔfleQ* carrying plasmid pMQ70*fleQ*	This study
Δhcp1	PAO1 containing a transposon insertion	Jacobs et al., 2003
Δhcp1+hcp1	PAO1-*Δhcp1* carrying plasmid pAK1900*hcp1*	This study
ΔclpV1	PAO1 containing a transposon insertion	Jacobs et al., 2003
ΔclpV1+clpV1	PAO1-*ΔclpV1* carrying plasmid pMQ70*clpV1*	This study
K. pneumoniae		
KP	*K. pneumoniae* ATCC 700603 carrying	This study
KP-GFP	KP harbors pMMG express GFP	This study
S. aureus		
SA	*S. aureus* ATCC 25923	ATCC
**Other strains**		
Corynebacterium argentoratense	Isolated from COPD patient	This study
Escherichia coli	Isolated from COPD patient	This study

Supplementary Table 2. Primers used in this study.

Primers	Sequence (5’ to 3’)
RhlR-F	CCCAAGCTTGTGCTGGCATAACAGATAGGGT
RhlR-R	CGGGGTACCGTCGGAGGACATACCAGCACA
FleQ-F	CGGGGTACCATGGCTTTGTGCCGTTACTT
FleQ-R	CCCAAGCTTGAAAATCAAAGCGTTGCGAA
Hcp1-F	CCCAAGCTTATGGCTGTTGATATGTTCATC
Hcp1-R	CGGGGTACCTCAGGCCTGCACGTTCTGGC
ClpV1-F	CGGGGTACCATGAGTGAGATCAGTCGCGTT
ClpV1-R	CCCAAGCTTCCTTCTACTGCTCTGCGTGTC
16S*whole*-M13F	TGTAAAACGACGGCCAGTAGAGTTTGATCCTGGCTCAG
16S*whole*-M13R	CAGGAAACAGCTATGACAAGGAGGTGATCCAGCCGCA
LasR*whole*-F	ACGCTGCGGTCTATTGTTA
LasR*whole*-R	ATCTCGCCCAGCAGTTTT
LasR*rt*-F	CTTCATCGTCGGCAACTAC
LasR*rt*-R	GTCTGGTAGATGGACGGTTC
LasB*rt*-F	ATCGGCTACGACATCAAGAAGG
LasB*rt*-R	CCGCTGTTGTAGTTGCTGGTG
RhlR*rt*-F	GCTCCTCGGAAATGGTGGT
RhlR*rt*-R	GGAAAGCACGCTGAGCAAAT
RhlA*rt*-F	AACGAGACCGTCGGCAAATA
RhlA*rt*-R	GCTCCAGGCAAGCCAAGTAG
FlgB*rt*-F	CGACAGAGCACTCGGTATCCA
FlgB*rt*-R	AAGGTGAAGGACGCCTGGAA
FlgC*rt*-F	GCCAACGGCTACGTGTATTACC
FlgC*rt*-R	ATCTGTTTGGCGGTATTCATCA
FlgD*rt*-F	AGTTGAAGAACCAGGACCCGA
FlgD*rt*-R	CCATGCTCTTGTTCAGCGACT
Hcp1*rt*-F	CAAGACTCACGCCGAGGAAA
Hcp1*rt*-R	TTCGCCTGCGGATAGTGCT
16S*rt*-F	GGACGGGTGAGTAATGCCTA
16S*rt*-R	CGTAGGAGTCTGGACCGTGT

Supplementary Table 3. Characterization of *lasR* mutants of *P. aeruginosa* during *in vitro* evolution.

Variants	Cycle	Mutation[1]	Change[2]	Protease[3]
PA[4]				
lasR1	14	C→T (+675)	Val→ Ile	–
lasR2	28	G→A (+541)	Glu → Lys	–
lasR3	28	G→C (+127)	Asp → His	–
Mixture[5]				
lasR4	21	G→A (+541)	Glu → Lys	–
lasR5	28	T→C (+155)	Ile → Thr	–
PASA[6]				
lasR6	21	G→C (+127)	Asp → His	–
PAKP[7]				
lasR7	21	G→A (+541)	Glu → Lys	–
ΔlasR	N/A	Deletion	N/A	–
WT PAO1	N/A	None	N/A	+

N/A, not applicable.
[1] Sites of nucleotide mutation relative to translational start site of the *P. aeruginosa lasR* gene.
[2] Amino acid changes relative to the LasR protein sequence of *P. aeruginosa*.
[3] '+' indicates the presence and '–' indicates the absence of a halo on M9-casein (0.5%) plates.
[4] Evolution of mono-cultured *P. aeruginosa.*
[5] Evolution of co-cultured *P. aeruginosa*, *S. aureus*, and *K. pneumonia*.
[6] Evolution of co-cultured *P. aeruginosa* and *S. aureus*.
[7] Evolution of co-cultured *P. aeruginosa* and *K. pneumonia*.

Supplementary Tables

Supplementary Dataset 1. Differentially expressed QS-regulated genes of *P. aeruginosa* between mono-culture of 1 and 21 days. FDR<0.05.

Supplementary Dataset 2. Differentially expressed genes of *P. aeruginosa* between mono-culture and co-culture with *K. pneumoniae* and *S. aureus* for 21 days. FDR<0.05.

Supplementary Dataset 3. Differentially expressed genes of *P. aeruginosa* between different culture conditions. 3 Mixture, co-culture of *P. aeruginosa*, *K. pneumoniae* and *S. aureus* for 21 days. PASA, co-culture of P. aeruginosa and S. aureus for 21 days. FDR<0.05.

Supplementary Dataset 4. Differentially expressed genes of *P. aeruginosa* between different culture conditions. 3 Mixture, co-culture of *P. aeruginosa*, *K. pneumoniae* and *S. aureus* for 21 days. PAKP, co-culture of P. aeruginosa and K. pneumoniae for 21 days. FDR<0.05.

Supplementary Dataset 5. Differentially expressed genes of *P. aeruginosa* between mono-culture and co-culture with *S. aureus* for 21 days.

Supplementary Dataset 6. Differentially expressed genes of *P. aeruginosa* between mono-culture and co-culture with *K. pneumoniae* after 21 days. FDR<0.05.

Supplementary Dataset 7. Expression of flagellar genes when *P. aeruginosa* was cultured under different culture conditions.

Supplementary Dataset 8. Expression of T6SS-related genes when *P. aeruginosa* was cultured under different culture conditions.

Supplementary Dataset 9. Differentially expressed genes of *P. aeruginosa* between mono-culture and co-culture with *K. pneumoniae* after 1 day.
